# Tocilizumab for refractory Castleman’s disease with paraneoplastic pemphigus and Behçet’s disease

**DOI:** 10.1186/1546-0096-10-S1-A95

**Published:** 2012-07-13

**Authors:** Ana Beatriz Vargas, Beatriz M Trope, Blanca ERG Bica

**Affiliations:** 1 Hospital Universitário Clementino Fraga Filho - Universidade Federal do Rio de Janeiro, Rio de Janeiro, Brazil

## Purpose

Castleman’s disease (CD) is an uncommon lymphoproliferative disorder, eventually complicated with paraneoplastic pemphigus (PNP) and pulmonary involvement. The association of Behçet and CD is very rare and the outcome is worse when PNP is present.

## Methods

We describe a 17 yo female patient with Behçet’s syndrome and CD. She was affected by a 4-month history of severe oral and genital ulcers, bilateral uveitis and abdominal pain. A CT scan of the abdomen showed the presence of a retroperitoneal mass (9,4 x 9,0 x 7,4 cm). On the basis of histological and immunohistological findings, a diagnosis of CD, hyaline vascular type, was formulated. There was no lymphadenopathy, fever or anemia. One month later, oral ulcers turned into desquamative plaques, Nikolsky sign was observed and an oral biopsy diagnosed paraneoplastic pemphigus. Ca 125 and Ca 19-9 levels were elevated. The patient developed an acute pleuritic pain with dyspnea, image exams revealed pneumomediastinum, pneumothorax and subcutaneous emphysema, and a bronchoscopy revealed a tracheal lesion suggestive of pemphigus. No signs of infection were present and pulses of methylprednisolone were prescribed, with good initial response. After the surgical excision of the retroperitoneal mass (June, 2010), she developed progressive dyspnea and a pulmonary function test revealed a mixed respiratory disorder. A thoracic CT scan showed patchy areas of ground-glass opacity. Thromboembolism was excluded. She was treated with high doses of prednisolone, colchicines, cyclosporine 200mg/day, with no response. Extensive mucosal erosions were still present, the tumor markers kept on rising and she presented a severe pulmonary restriction. We decided to initiate Tocilizumab EOW associated with moderate dose of prednisone.

## Results

After 5 months of Tocilizumab use, the oral lesions were considerably better, but there were only a subtle increase of forced vital capacity and little (if some) pulmonary clinical response. A PET scan failed to show new tumor lesions. Although most patients with the benign type of CD have shown remission of PNP upon excision of the tumor, our patient showed severe pulmonary involvement despite of IL6 blockage treatment.

## Conclusion

Although the association of Behçet’s disease and pemphigus is very intriguing, CD should always be investigated in a young patient with PNP. Localized hyaline-vascular CD is generally benign, fully responding to surgery. However, the presence of PNP with pulmonary involvement carries a poor prognosis.

## Disclosure

Ana Beatriz Vargas: None; Beatriz M. Trope: None; Blanca E. R. G. Bica: None.

